# Exploring the adverse events of Oxford–AstraZeneca, Pfizer-BioNTech, Moderna, and Johnson and Johnson COVID-19 vaccination on Guillain–Barré Syndrome

**DOI:** 10.1038/s41598-024-66999-7

**Published:** 2024-08-13

**Authors:** Sultan Ayoub Meo, Narmeen Shaikh, Farah Adnan Abukhalaf, Anusha Sultan Meo

**Affiliations:** 1 Department of Physiology, College of Medicine, King Saud University, Riyadh, Saudi Arabia; 2 College of Medicine, King Saud University, Riyadh, Saudi Arabia; 3 The School of Medicine, Medical Sciences and Nutrition, University of Aberdeen, Aberdeen, Scotland, UK

**Keywords:** COVID-19 pandemic, Vaccines, Oxford–AstraZeneca, Pfizer-BioNTech, Moderna, Johnson and Johnson, Neurological events, Guillain–Barré Syndrome, GBS, Microbiology, Neuroscience, Physiology, Health care

## Abstract

The vaccination against Severe Acute Respiratory Syndrome Coronavirus 2 (SARS-CoV-2) is an important public health strategy to prevent people from the pandemic. Vaccines are a game-changing tool, it is essential to understand the adverse events after COVID-19 vaccination. This study explored the adverse events of COVID-19 Vaccination Oxford–AstraZeneca, Pfizer-BioNTech, Moderna, Johnson and Johnson on Guillain–Barré Syndrome (GBS). In this study, initially 128 documents were identified from the databases, including Pub-Med, Web of Science-Clarivate Analytics, Scopus, and Google Scholar. The articles on COVID-19 vaccination and GBs were searched using the keywords “SARS-CoV-2, COVID-19, Vaccination, and Guillain Barré Syndrome, GBS”, finally, 16 documents were included in the analysis and synthesis. After administering 1,680,042,214 doses of COVID-19 vaccines, 6177 cases were identified with 10.5 cases per million vaccine doses. A significant positive risk was found between COVID-19 vaccine administration and GBS with a risk rate of RR 1.97 (95% CI 1.26–3.08, p = 0.01). The mRNA vaccines were associated with 2076 cases, and 1,237,638,401 vaccine doses were linked with 4.47 GBS events per million vaccine doses. The first dose of the m-RNA vaccine was associated with 8.83 events per million doses compared to the second dose with 02 events per million doses. The viral-vector vaccine doses 193,535,249 were linked to 1630 GBS cases with 11.01 cases per million doses. The incidence of GBS after the first dose was 17.43 compared to 1.47 cases per million in the second dose of the viral-vector vaccine. The adverse events of the Oxford–AstraZeneca vaccine were linked to 1339 cases of GBS following 167,786,902 vaccine doses, with 14.2 cases per million doses. The Oxford–AstraZeneca vaccine significantly increased the risk of GBS RR: 2.96 (95% CI 2.51–3.48, p = 0.01). For the Pfizer-BioNTech vaccine, there were 7.20 cases per million doses of the vaccine, and no significant association was identified between the Pfizer-BioNTech vaccine and GBS incidence RR: 0.99 (95% CI 0.75–1.32, p = 0.96). Moderna vaccine was related with 419 cases of GBS after administering 420,420,909 doses, with 2.26 cases per million doses. However, Johnson and Johnson's vaccination was linked to 235 GBS after 60,256,913 doses of the vaccine with 8.80 cases per million doses. A significant association was seen between the risk of GBS and Ad.26.COV2. S vaccine, RR: 2.47 (95% CI 1.30–4.69, p < 0.01). Overall, a significant association was seen between the COVID-19 vaccines and the risk of GBS. The incidence of GBS was higher after the first dose compared to GBS cases per million in the second dose.

## Introduction

The outbreak of “Severe Acute Respiratory Syndrome Coronavirus 2 (SARS-CoV-2), also known as the COVID-19 Pandemic”, caused a substantial adverse impact on public health, the economy, psychological well-being, and the loss of millions of human lives globally^1,2^. The COVID-19 pandemic caused panic conditions among the public due to swift transmission, the severity of diseases, emerging and re-emerging characteristics, and various variants of the virus, further enhancing the alarming situation worldwide. Many countries have executed strict measures to mitigate the COVID-19 pandemic. These policies include wearing face masks, social distancing, quarantine, lockdowns, travel restrictions, and vaccination^3^.

Vaccination is the most significant medical intervention against the COVID-19 pandemic and is the best strategy for inducing immunity and preventing diseases. Vaccines are convenient measures to improve healthcare outcomes and healthy life expectancy by preventing and controlling infectious diseases at regional and global levels^4,5^.

Since December 2020, COVID-19 vaccines have developed great hope to fight against the deadly pandemic. Many vaccines were introduced worldwide, however, a few vaccine types which were frequently used include “viral vector vaccines, COVID-19 messenger RNA (mRNA) based vaccines, inactivated or attenuated virus vaccines, and protein-based vaccines”^6,7^. These vaccines significantly minimised the pandemic, but at the same time, the literature highlighted the rising risk of neurological adverse events such as “acute disseminated encephalomyelitis, transverse myelitis, aseptic meningitis, and myositis.” The mechanism behind these neurological complications is due to molecular mimicry, neurotoxicity, and aberrant immune reactions, which have been recognized to describe the vaccines' allied nervous system problems^8,9^.

The vaccines are highly beneficial for the prevention of the pandemic, and the vaccination campaign against the COVID-19 pandemic is a significant public health strategy to eliminate the disease burden and prevent the pandemic^10^. About 13.57 billion doses have been managed globally, with 7.03 million daily doses^11^. Despite the life-saving role of vaccination against SARS-CoV-2 and COVID-19, vaccines are not completely free from complications and concerns about vaccine-related side effects have grown worldwide^12^. Safe and effective vaccines are a game-changing tool, but it is also essential to understand the adverse neurological events such as Guillain Barré Syndrome (GBS) associated with COVID-19 vaccination. Recent reports on COVID-19 vaccinations and GBS are leading to regulatory, clinical and public health concerns. However, the literature is lacking in establishing the links between frequently used COVID-19 vaccines and GBS. The study findings may provide appropriate information and better understanding to healthcare officials and policymakers while establishing public health strategies. Therefore, this study aimed to investigate the adverse events of COVID-19 Vaccination Oxford–AstraZeneca, Pfizer-BioNTech, Moderna, Johnson and Johnson on Guillain Barré Syndrome (GBS).

## Methods

The study was conducted in the “Department of Physiology, College of Medicine, King Saud University, Riyadh, Saudi Arabia.”

### Data extraction

The data was composed from the relevant databases regarding the adverse events of “Oxford–AstraZeneca (ChAdOx1nCoV-19/ChAdOx1-S), Pfizer-BioNTech (BNT162b2 mRNA), Moderna (mRNA-1273), and Johnson and Johnson (Janssen- Ad.26.COV2. S), COVID 19 Vaccination on Guillain–Barré Syndrome (GBS)”. The required information was recorded from various databases, including “PubMed, Web of Science, Google Scholar, World Health Organization (WHO), and Centers for Disease Control and Prevention (CDC).” The key terms used to record the information include: “Coronavirus, SARS-CoV-2, COVID-19 pandemic, vaccines, adverse events, complications, Oxford–AstraZeneca (ChAdOx1nCoV-19/ChAdOx1-S), Pfizer-BioNTech (BNT162b2 mRNA), Moderna (mRNA-1273), and Johnson and Johnson (Janssen-Ad.26.COV2. S), vaccine, Moderna vaccine”. The information was gathered without specific limitations on publication status or study design; however, the English language of publication was imposed. Two research team members reviewed the literature, and their findings were entered. After that, another co-author rechecked the literature and their findings. From the 128 identified documents, we included 16 documents in the analysis, synthesis, and discussion.

### Inclusion and exclusion criteria

The documents included in this study must be original and data containing the information on adverse events of “Oxford–AstraZeneca (ChAdOx1nCoV-19/ ChAdOx1-S), Pfizer-BioNTech (BNT162b2 mRNA), Moderna (mRNA-1273), and Johnson and Johnson (Janssen- Ad.26.COV2. S) COVID 19 Vaccination on Guillain–Barré Syndrome (GBS)”. The vaccines other than GBS adverse events were excluded from the study. Moreover, we included original studies, however, brief communications, letters to the editor, case reports, and review articles were excluded from the study. The information was gathered without specific limitations on publication status or study design; however, the English language of publication was imposed.

### Statistical analysis

The statistical analyses were performed using STATA18 and RStudio version 4.3.2. From all these studies, relevant information was retrieved, such as the total number of GBS events, total population and total COVID-19 doses administered and estimated total number of events per million doses using the formula Events/Total Doses given*1,000,000. We pooled the results using a random effect model. We also calculated the O/E (Observed/Expected) ratio with corresponding 95% confidence intervals. The OE analysis involved a comparison of the number of GBS reports after the vaccine with the expected number of cases in each study. The results for each study were pooled using a random effects model. O/E incident ratio of > 1 was considered elevated, and < 1 was considered lower. Some studies also mentioned risk ratios (RR), which were extracted and pooled to explore the link between GBS and COVID-19 vaccine using the Mantel–Haenszel method^13^. This was only done for studies that reported RR, and the number of studies was three or more. We analyzed the association overall regardless of the vaccine technology used. We also sub-grouped the studies into 1st vs 2nd dose, m-RNA vs viral vector vaccines and finally, specific vaccine types. A p-value p < 0.05 was considered significant for all analyses. The “Cochrane chi-squared test (Chi^2^) was used to evaluate heterogeneity among articles; a *p*-value < 0.05 indicates the existence of heterogeneity. *I*^*2*^ value was calculated. *I*^*2*^ is a measure of heterogeneity and a method for calculating the associated 95% CI. *I*^*2*^ expresses the proportion of variability in a meta-analysis. *I*^*2*^ values ≥ 50% and *p* < 0.05 indicated a moderate to high degree of heterogeneity among pooled studies^14^. Egger's test was performed wherever appropriate to evaluate publication bias, further assessed by visually examining the symmetry in funnel plots. A leave-one-out sensitivity analysis was performed to check the reliability of some studies.

### Ethics approval

This study was exempted from ethical approval or informed consent because data were obtained from publicly available sources.

## Results

We found 16 studies that reported the association between the incidence of GBS after COVID-19 administration. Some studies did not report the total sample size. However, from the 12 that did, the estimated population is 213,205,062. Within these studies, 1,680,042,214 doses of COVID-19 vaccines were given. 6177 cases of GBS were reported; among these, around 55% were males. The studies spanned five continents: five studies included the population from the UK and/or European Union, two from the USA and one each from Mexico, Australia, Jordan, South Korea, Singapore, and South Africa. From all studies, we analysed information on the four primary vaccines (Table 1).

**Table 1:** The studies included with GBS events after COVID-19 vaccinations.

Author, Year, Country	Study type	Vaccine	Age, total vaccinated population, total number of doses	Number of GBS cases
Patone et al., 2021, England^15^	Self-controlled case series study	ChAdOx1nCoV-19 BNT162b2 mRNA	Mean Age: 55 years Total: 32,552,534	187 events 28 days after the first dose
Hanson et al. 2022, USA^16^	Cohort	mRNA-1273 Ad.26.COV2. S	Mean Age: 46.5 years, Total: 7 894 989, Doses: 15 120 073	31 cases between 1–42 days after the last dose
Le Vu et al., 2023, France,^17^	Self-controlled case series study	ChAdOx1-S & Ad26.COV2-S	Median Age: 57 Total: 58,530,770 Doses: 88.8% with at least first dose	2229 cases after one dose of the vaccines between 1–42 days
Morciano et al., 2024, Italy^18^	Self-controlled case series study	ChAdOx1-S BNT162b2 mRNA-1273 Ad26.COV2-S	Median age: 56 Total: 15,986,009 Doses: 27,038,926	287 new cases of GBS after the first or second dose
Walker et al., 2022, UK^19^	Self-controlled case series	ChAdOx1 BNT162b2	Median Age: 62 Total: 13,512,593	800 cases of GBS between 1–42 days after first dose
Atzenhoffer et al., 2022, European Union, USA, Ecuador, Iceland, Norway, Switzerland, Uruguay^20^	Cohort study	ChAdOx1-S BNT162b2 mRNA-1273 Ad26.COV2-S	Median Age: 57 Total doses of mRNA vaccine: 770,856,446 Total doses of adenovirus vaccine: 95,246,095	1232 cases for all mRNA-based vaccines within 1–42 days 610 cases for all adenovirus-based vaccines within 1–42 days
García-Grimshaw, 2022, Mexico^21^	Retrospective study	ChAdOx1nCoV-19 BNT162b2 mRNA-1273 Ad26.COV2-S	Median Age: 44 Doses: 81,842,426	97 cases within 42 days after the last dose
Osowicki et al., 2022, Australia^22^	Case series	ChAdOx1-S BNT162b2	Median Age: 65 Total: 6.6. million Doses: 10,613,528	41 cases within 42 days after the last dose
Keh et al., 2023, England^23^	Population-based study	ChAdOx1nCoV-19 BNT162b2 mRNA-1273	Age: 18 + years Total: 55,980,000 Doses: 32.1 million	198 cases within 6 weeks of the last dose
Woo et al., 2021, USA^24^	Retrospective study	Ad26.COV2. S	Age: > 18 years Doses: 13 209 858	123 cases within 42 days after the last dose
Abara et al., 2023, USA^25^	Cohort Study	BNT162b2 mRNA-1273 Ad26.COV2-S	Median Age: 55 Dose: 487,651,785	253 cases within 42 days after the last dose
Abdel-Qader et al., 2022, Jordan^26^	Prospective study	ChAdOx1nCoV-19 BNT162b2	Population: 658,428 Doses: 1,032,430	12 cases 14 days after the first or second dose
Ha et al., 2023, South Korea^27^	Prospective Regional Surveillance Study	m-RNA vaccine viral vector vaccine	Age: 86.8% of 13 million people, Total doses: 38,828,691	55 cases within 42 days after the first or second dose
Koh et al., 2021, Singapore^28^	Prospective study	BNT162b2 mRNA-1273	Median Age: 59 ys Total: 1,398,074 Doses: 65.5% of people got 2 doses, the rest 1 dose	2 cases with a median latency period of 4 days
Li et al., 2022, UK & Spain^29^	Population-based cohort & self-controlled case series	ChAdOx1nCoV-19 BNT162b2	Median Age: UK (48), Spain (47) Total: 8 330 497 got at least one dose	11 cases in the UK, 21 days after ChAdOx1nCoV-19 first dose. 5 cases in Spain 21 days after BNT162b2 first dose
Takuva et al., 2022, South Africa^30^	Open-label phase 3b implementation study	Ad26.COV2. S	Median age: 42 Total: 477,234 Dose: 477,234	4 cases within 28 days of a single vaccine dose

### Guillain–Barré Syndrome (GBS) incidence after COVID-19 vaccination irrespective of the vaccine types

**Figure 1:**
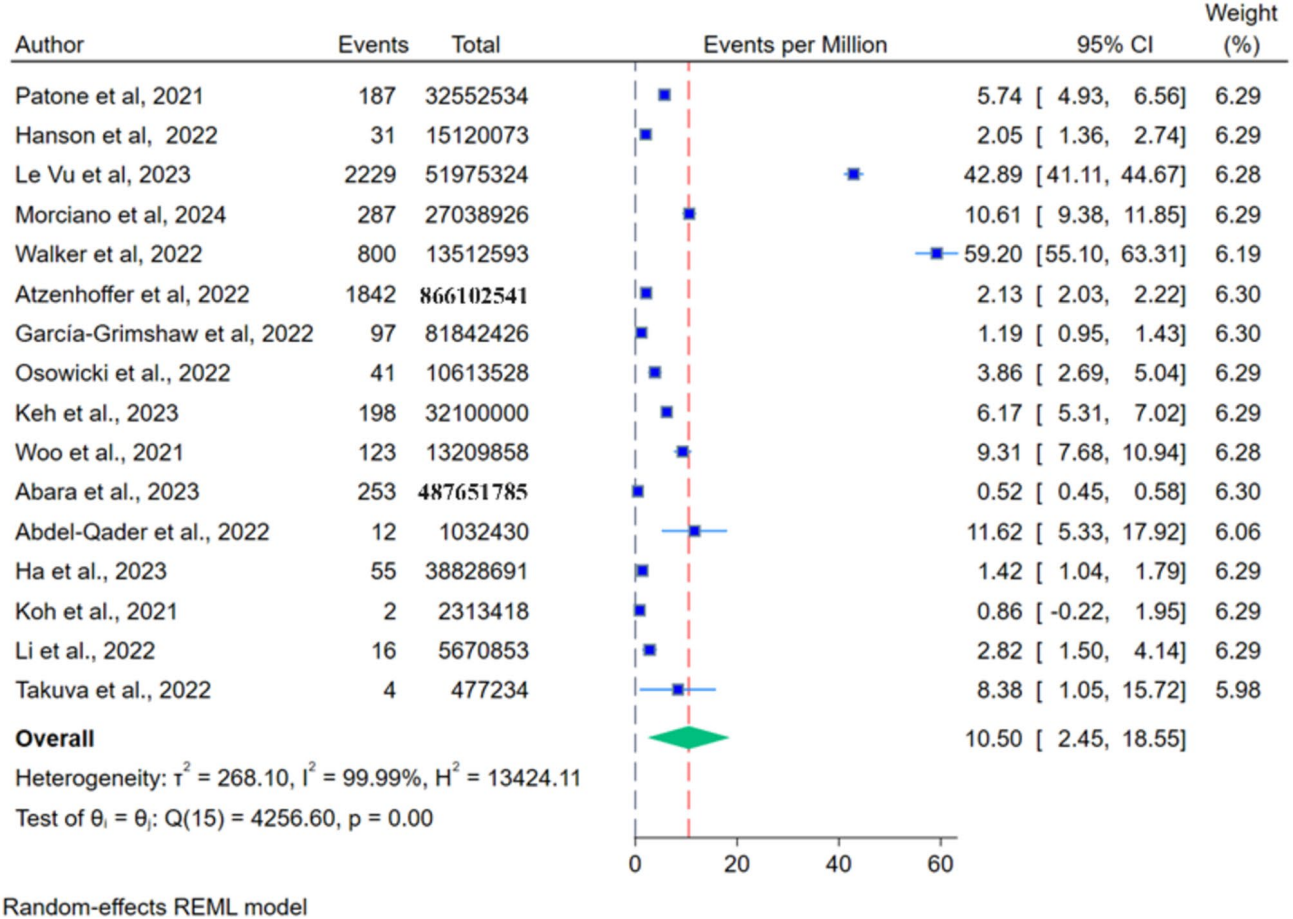
Forest plot for per million events for Oxford–AstraZeneca, Pfizer-BioNTech, Moderna, and Johnson and Johnson vaccines on Guillain–Barré Syndrome.

The overall analysis of adverse events of Oxford–AstraZeneca, Pfizer-BioNTech, Moderna, and Johnson and Johnson COVID-19 Vaccination on Guillain–Barré Syndrome (GBS) were analysed through events per million, O/E analysis and risk ratios from all relevant studies. Sensitivity analysis (leave-one-out) was done wherever appropriate and showed that no study significantly affected the effect size for each outcome. GBS events per million vaccine doses were estimated using data from 16 studies and pooled together (Fig. 1). A total of 6177 cases were seen after administering an estimated 1,680,042,214 doses of COVID-19 vaccines. The random effect model calculated 10.5 cases per million vaccine doses. Significant heterogenicity was present (Q: p = 0.00, I^2^ 99.99%).

**Figure 2:**
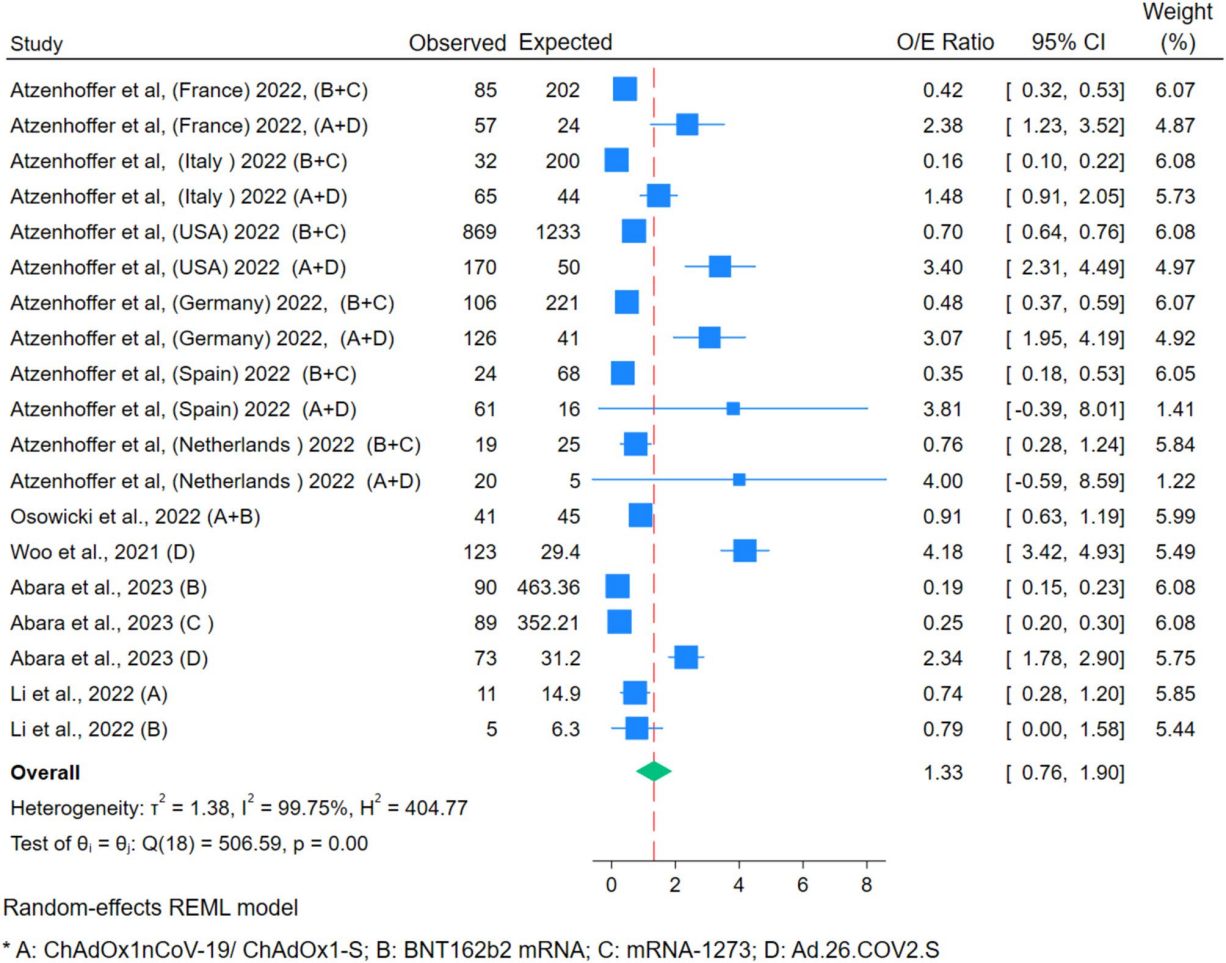
Forest Plot for O/E analysis for all vaccine Oxford–AstraZeneca, Pfizer-BioNTech, Moderna, and Johnson and Johnson vaccines on Guillain–Barré Syndrome.

O/E incident analyses of these four vaccine types (Fig. 2) were done in 5 studies comprising 19 cohorts (different vaccine types and different countries within the same study were used as separate data points). It was elevated: 1.33 (95% CI 0.76–1.90). A significant heterogenicity was present (Q: p = 0.00, I^2^ 99.7%). The funnel plot and Egger’s regression test showed the presence of publication bias.

**Figure 3:**
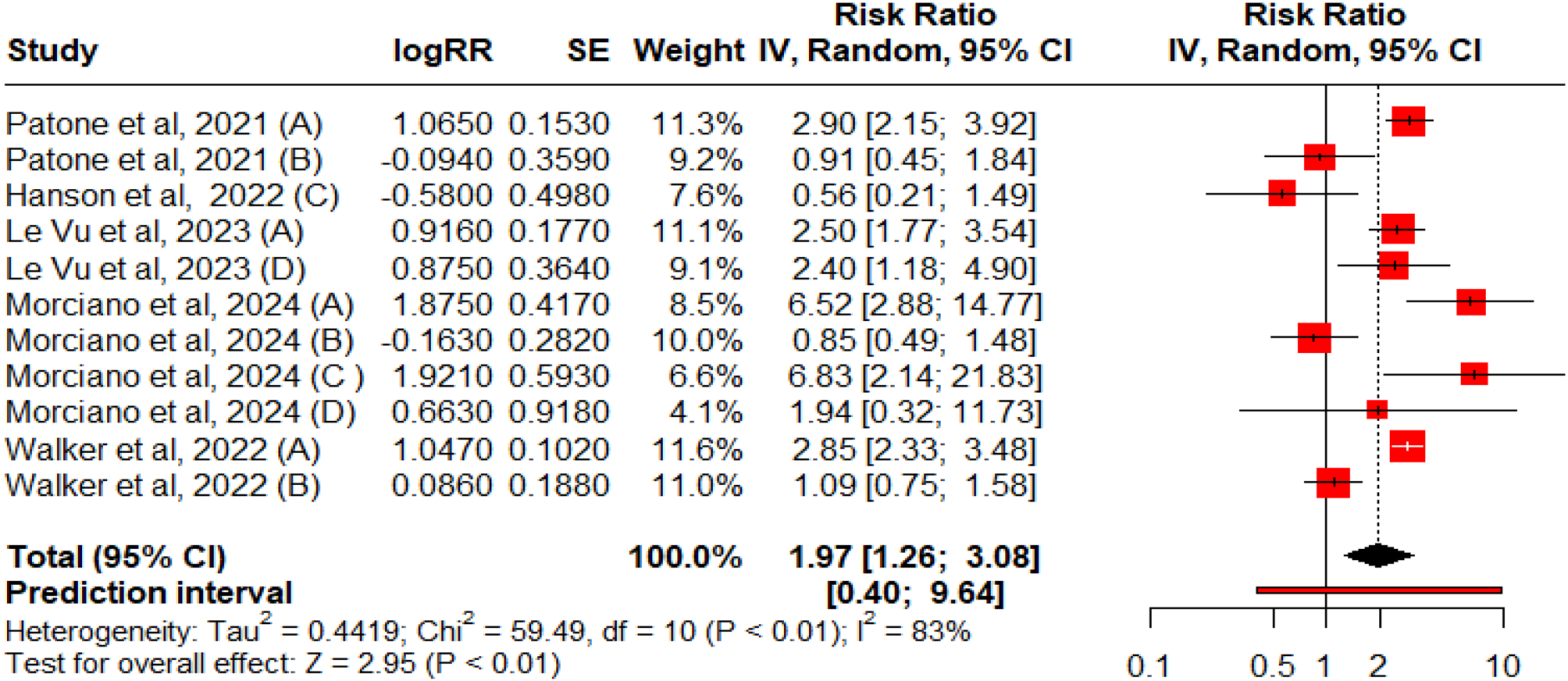
Forest Plot for RR for all vaccine vaccines Oxford–AstraZeneca, Pfizer-BioNTech, Moderna, and Johnson and Johnson vaccines on Guillain–Barré Syndrome. [*A: ChAdOx1nCoV-19/ ChAdOx1-S; B: BNT162b2 mRNA; C: mRNA-1273; D: Ad.26.COV2. S vaccines].

The risk ratios were reported by five studies (a total of 11 cohorts accounting for each vaccine type). A random model was used to pool the effect size (Fig. 3). A positive and significant association was seen between the risk of GBS and the administration of the COVID-19 vaccine with RR: 1.97 (95% CI 1.26–3.08, p = 0.01). Heterogenicity was significant (Q: 59.49, p = 0.01, I2 = 83%). The funnel plot indicated the presence of publication bias, but Egger’s regression test did not demonstrate the impact of bias.

### Association between m-RNA and viral-vector vaccine with GBS incidence mRNA vaccines

Our study analysed two types of m-RNA-based vaccines: the BNT162b2 mRNA vaccine and the mRNA-1273 vaccine. The mRNA vaccines accounted for 2076 cases, and 1,237,638,401 doses of the vaccines were given. The sensitivity analysis (leave-one-out) was done wherever appropriate and showed that no single study significantly affected the effect size for each outcome.

**Figure 4:**
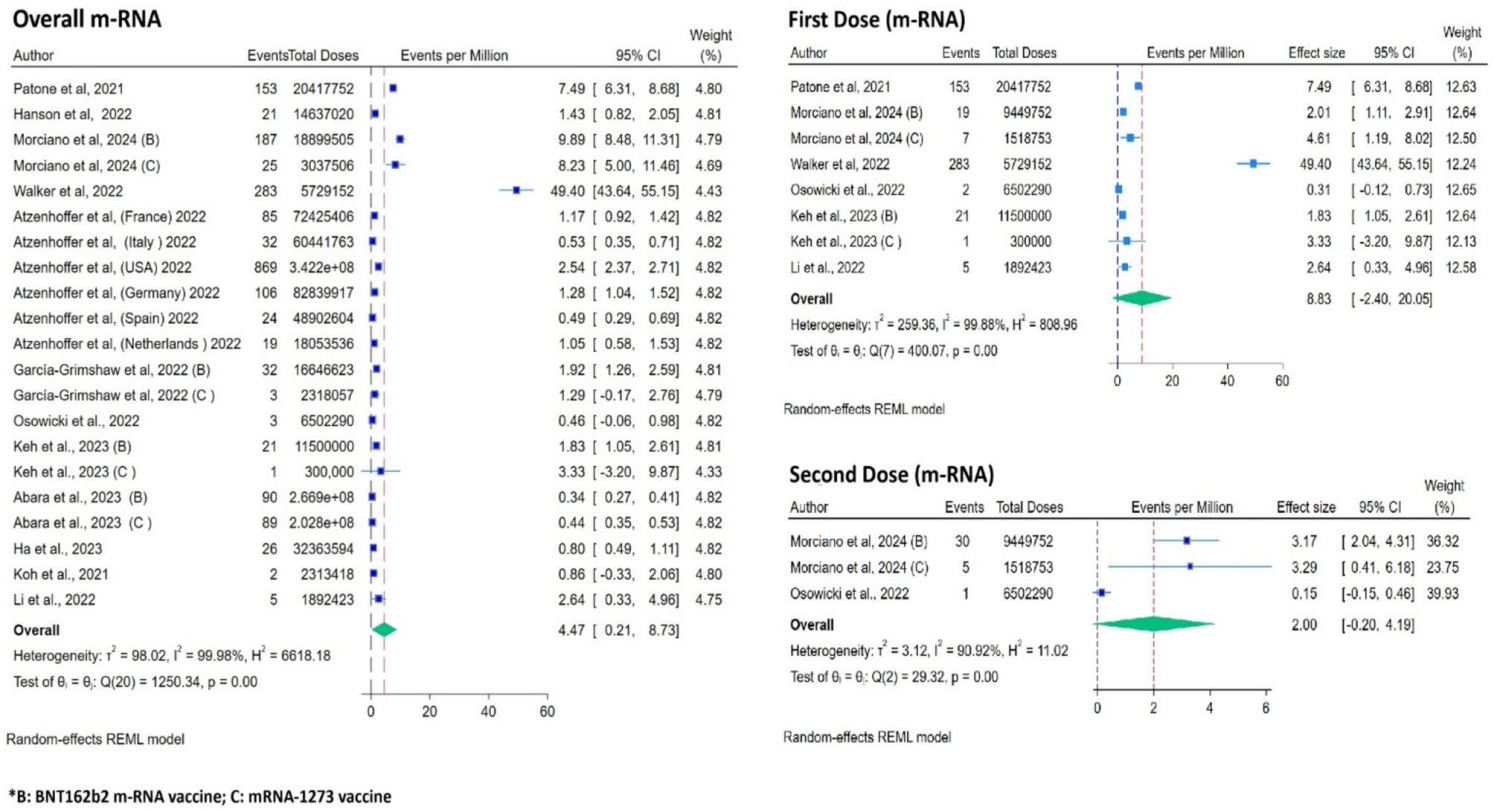
Forest Plots for GBS events per million doses of vaccines (overall, first dose and second dose of mRNA vaccine).

For analysis of GBS events per million dose (Fig. 4), 12 studies were included (21 cohorts as each vaccine and different countries within the same study were used as separate data points). It was estimated that m-RNA vaccines were associated with 4.47 GBS events per million vaccine doses. While considering the effect of the first and second doses on GBS, the first dose of the m-RNA vaccine per million dose rate was higher at 8.83 events per million compared to the second dose of the vaccine 2 events per million doses (Fig. 4; Table 2).

**Table 2:** Comparison between adverse events of viral vector vaccines (Oxford/AstraZeneca; Johnson and Johnson) and mRNA vaccines (Pfizer-BioNTech; Moderna) On Guillain–Barré Syndrome.

COVID-19 vaccines	Number of studies	Number of GBS cases	Number of vaccine doses	Cases per million doses of vaccine	Cases per million during first & second doses of Vaccines
Viral-vector vaccines (Oxford/AstraZeneca; Johnson and Johnson)	13	1630	193,535,249 doses of vaccine	11.01 GBS cases per million doses of vaccine	First dose: 17.43; Second dose: 1.47 cases per million
mRNA vaccines (Pfizer-BioNTech and Moderna)	12	2076	1,237,638,401 doses of vaccines	4.47 GBS per million doses of vaccines	First dose: 8.83 Second dose: 2 events per million doses

**Figure 5:**
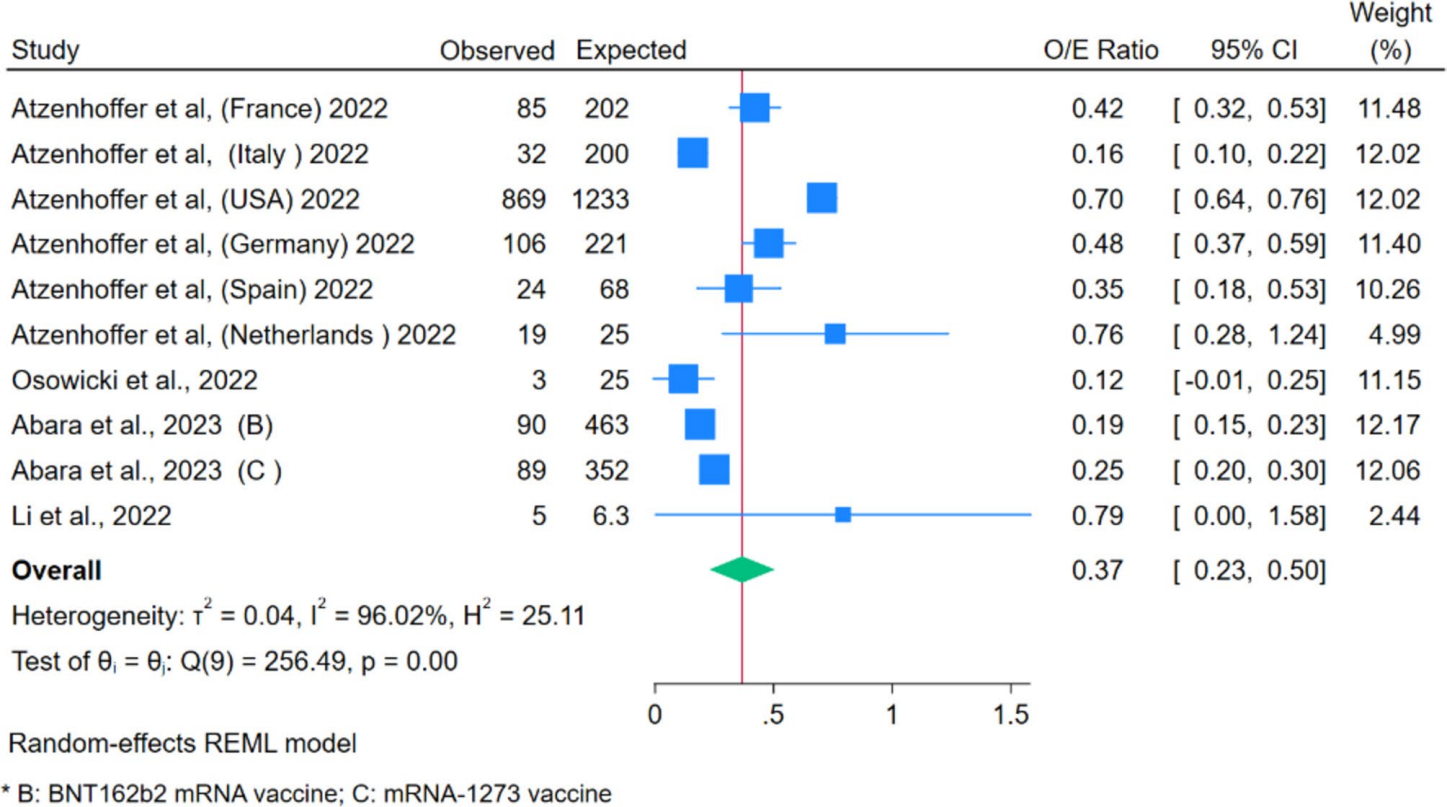
Forest Plot for O/E Ratio for m-RNA vaccines.

Observed/Expected ratios were also calculated for five studies (Fig. 5). Compared to the rates of GBS, the m-RNA vaccine did not increase the rates for GBS, with the pooled O/E ratio being 0.37 (95% CI 0.23–0.50). Heterogenicity was significant (Q: p = 0.00, I^2^ = 96%). The funnel plot showed the presence of publication bias, but Egger’s regression test did not (Supplementary Information).

### Viral vector-based vaccines

Our study analysed two viral vector-based vaccines (ChAdOx1nCoV-19/ ChAdOx1-S vaccine and Ad.26.COV2. S vaccine). The total number of viral-vector vaccine doses was approximately 193,535,249, which were linked to around 1,630 GBS cases. Sensitivity analysis (leave-one-out) was performed wherever appropriate and showed that no single study significantly affected the effect size for each outcome.

**Figure 6:**
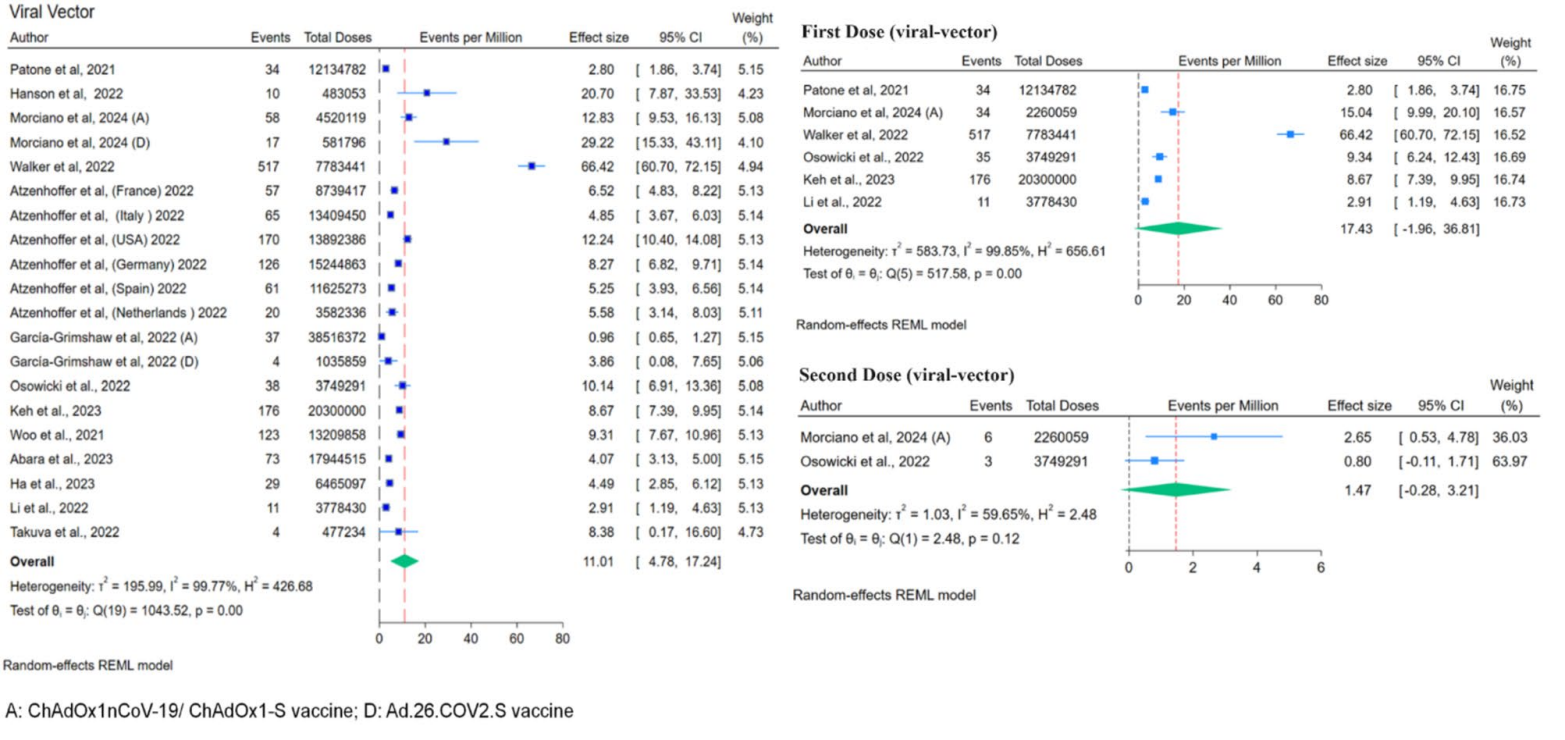
Forest Plots for GBS events per million doses (overall, first dose and second dose).

**Figure 7:**
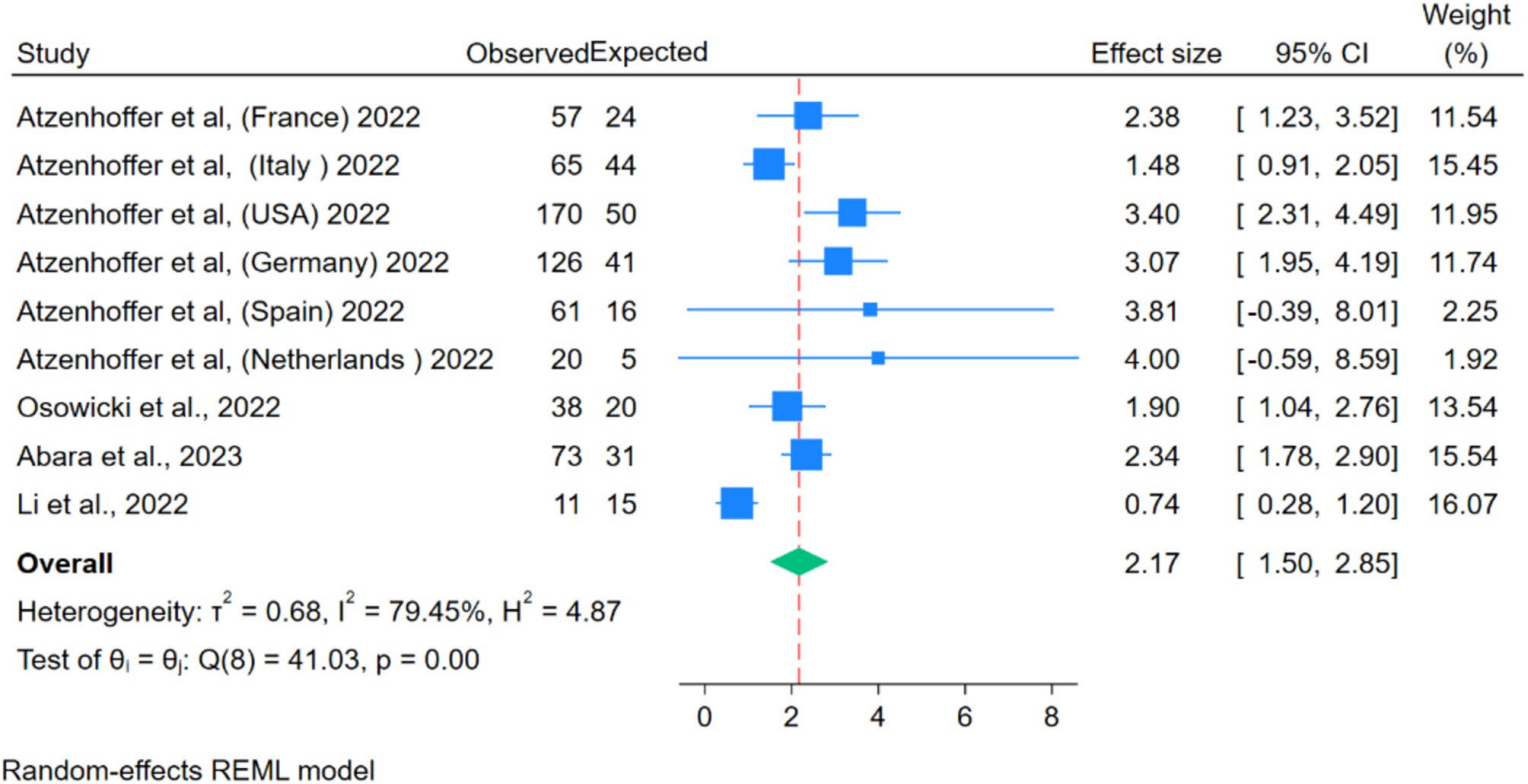
Forest Plot for observed to expected (O/E) ratio for viral vector vaccines.

The results presented in Fig. 6 revealed that viral-vector-based vaccines were associated with 11.01 GBS cases per million vaccine doses. Moreover, the incidence after the first dose was 17.43 events per million, higher than the second dose, 1.47 cases per million (Fig. 6). The observed to expected (O/E) ratio analysis was performed on four studies (9 cohorts as different countries within the same study were used as separate data points), and it showed that in comparison to background rates, there was an increase in GBS incidence after the administration of viral-vector based COVID-19 vaccines: 2.17 (95% CI 1.50–2.85) (Fig. 7). The funnel plot showed the presence of publication bias, but Egger’s regression test did not show the presence of publication bias.

### Specific vaccines and their association with GBS incidence

#### Oxford AstraZeneca (ChAdOx1nCoV-19/ChAdOx1-S) vaccine

**Figure 8:**
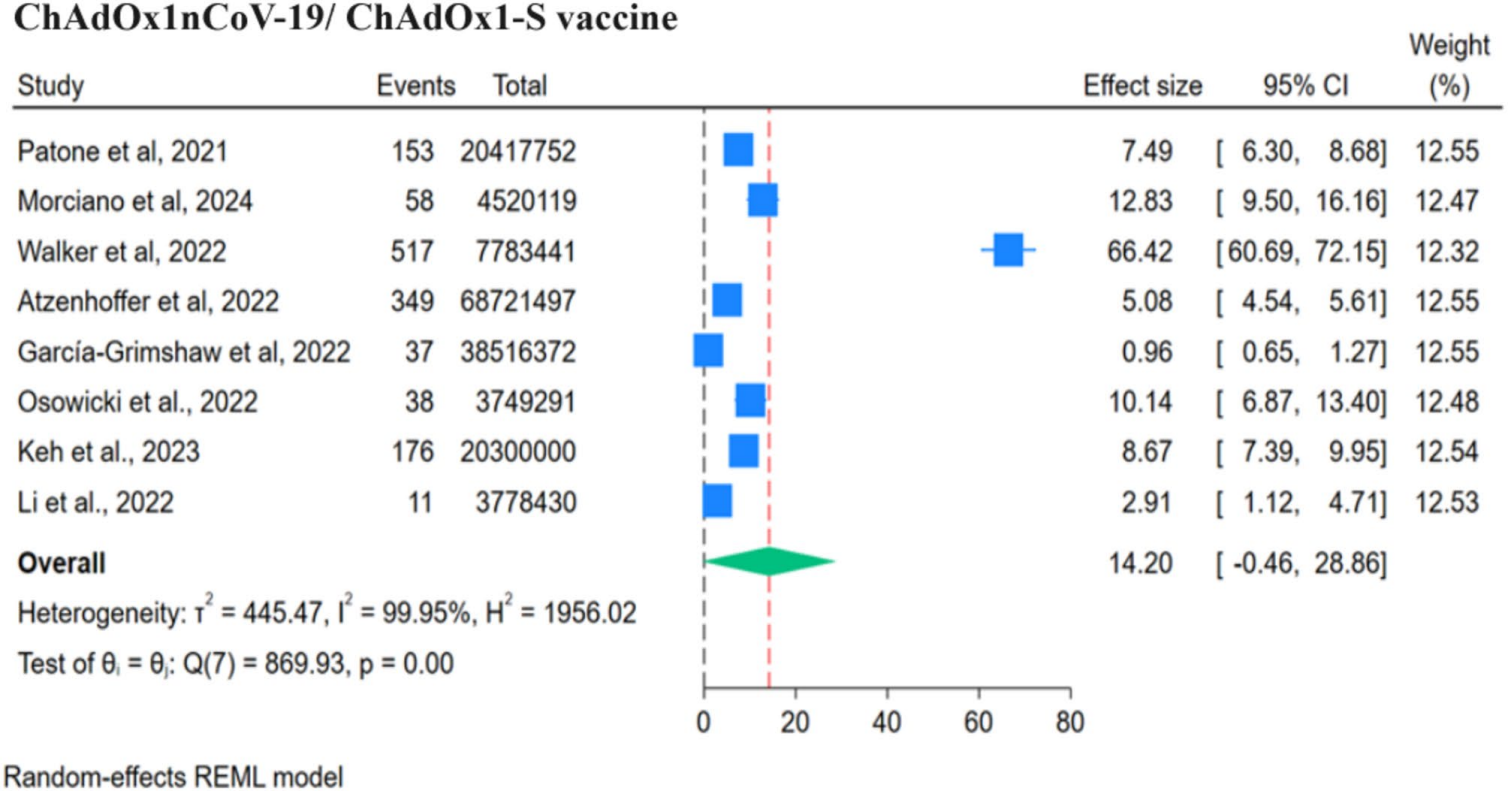
Forest Plots for GBS events per million doses of ChAdOx1nCoV-19 vaccine.

**Figure 9:**
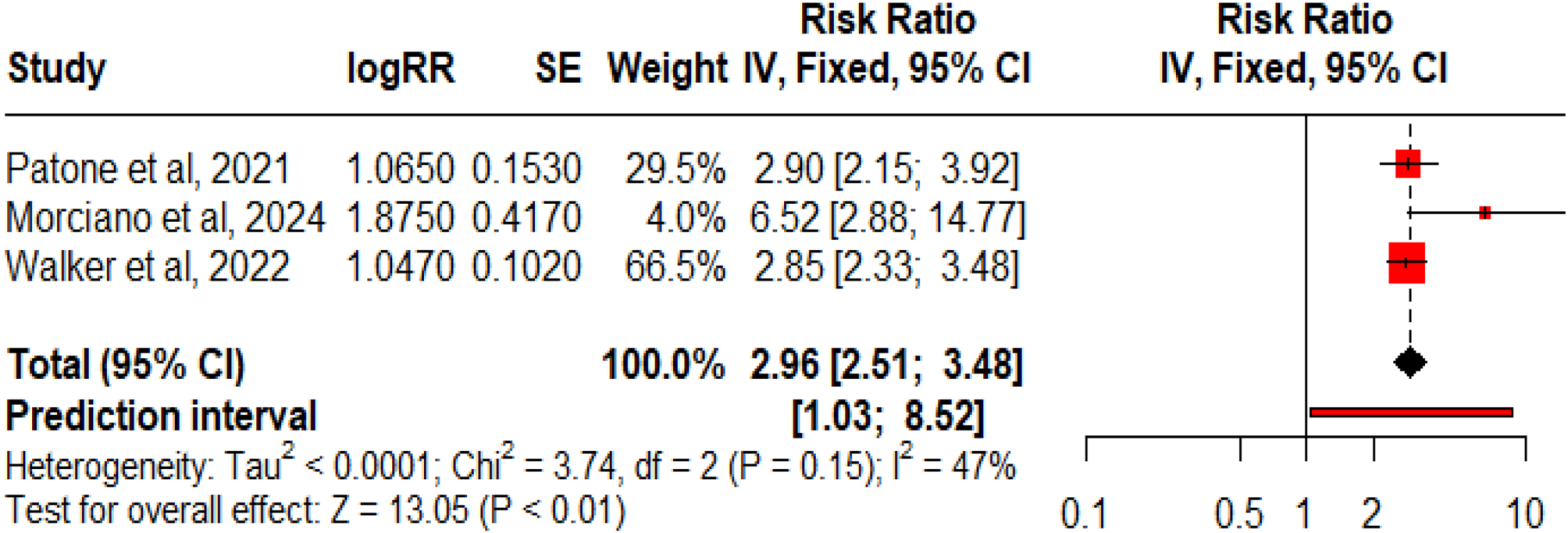
Forest Plot for risk ratio RR for ChAdOx1nCoV-19 vaccine.

Eight studies reported the ChAdOx1nCoV-19 vaccine and its association with GBS. In these studies, 1339 individuals reported GBS following around 167,786,902 vaccine doses that were given to the population. Figure 8 revealed that per million doses of the ChAdOx1nCoV-19 vaccine was associated with 14.2 cases of GBS. Three studies mentioned RR, which were pooled together (Fig. 9, Table 3) using a fixed effect model as heterogenicity was insignificant (Q: 3.74, p = 0.15, I^2^ = 47%). ChAdOx1nCoV-19/ChAdOx1-S vaccine is positively and significantly associated with the risk of GBS, RR: 2.96 (95% CI 2.51–3.48, p = 0.01). The funnel plot showed the presence of publication bias, but Egger’s regression test did not show the presence of publication bias.

**Table 3:** Adverse events of Oxford–AstraZeneca, Pfizer-BioNTech, Moderna, and Johnson and Johnson COVID-19 Vaccination on Guillain–Barré Syndrome.

COVID-19 vaccines	Number of studies	Number of GBS cases	Number of vaccine doses	Cases per million doses of vaccine	Risk ratio and significance level*
Viral-vector vaccines					
Oxford/AstraZeneca	8	1339	167,786,902	14.2 cases	RR: 2.96 (95% CI 2.51–3.48, p < 0.01)
Johnson and Johnson	7	235	60,256,913	8.80 cases	RR: 2.47 (95% CI 1.30–4.69, p < 0.01)
mRNA vaccines					
Pfizer-BioNTech,	10	1609	916,053,583	7.20 cases	RR: 0.99 (95% CI 0.75–1.32, p = 0.96)
Moderna	6	419	420,420,909	2.26 cases	––

*Pooled Risk Ratio is only for the studies that reported it.

### Pfizer-BioNTech (BNT162b2 mRNA) vaccine

**Figure 10:**
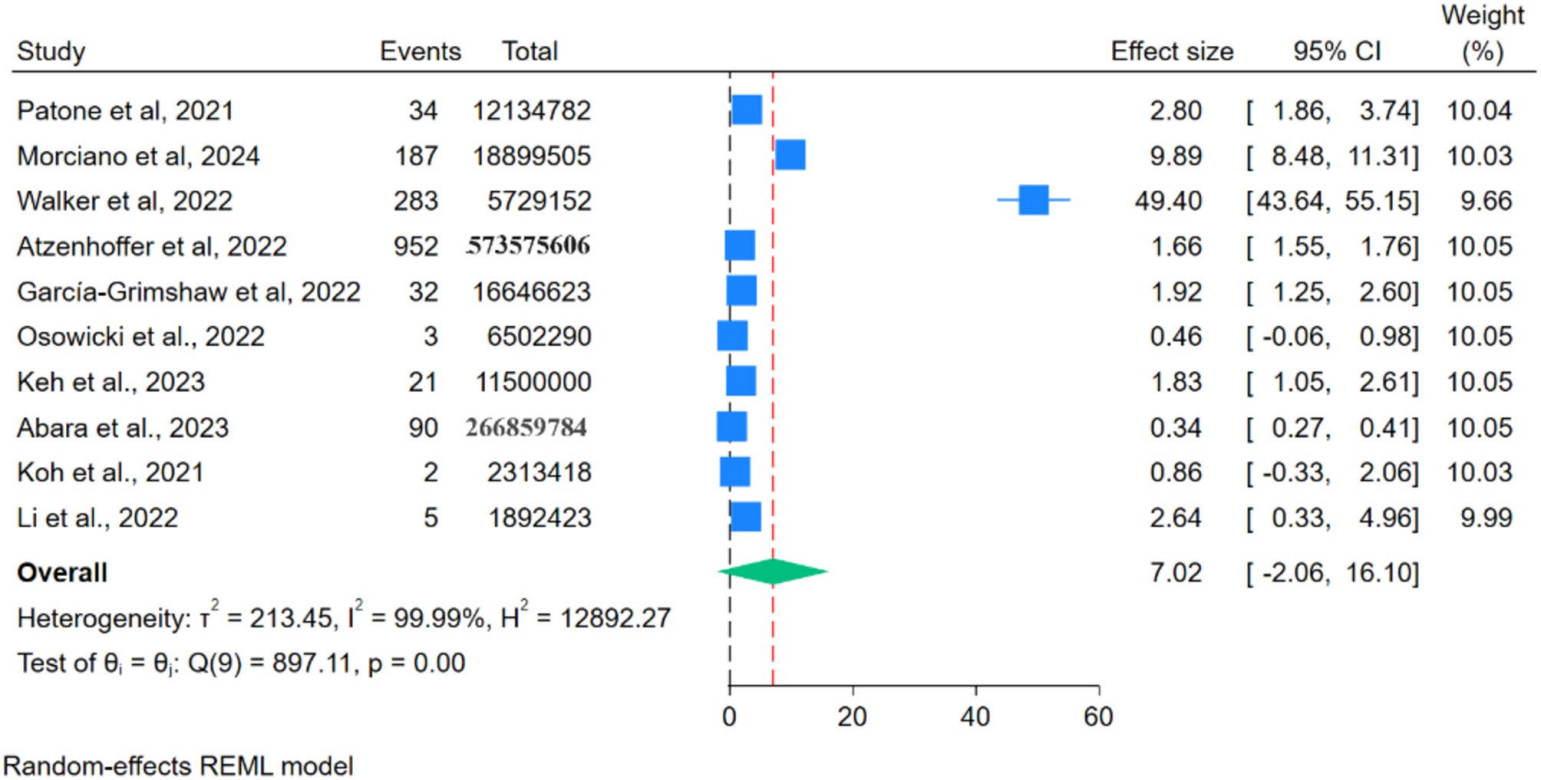
Forest Plots for GBS events per million doses of BNT162b2 mRNA vaccine.

**Figure 11:**
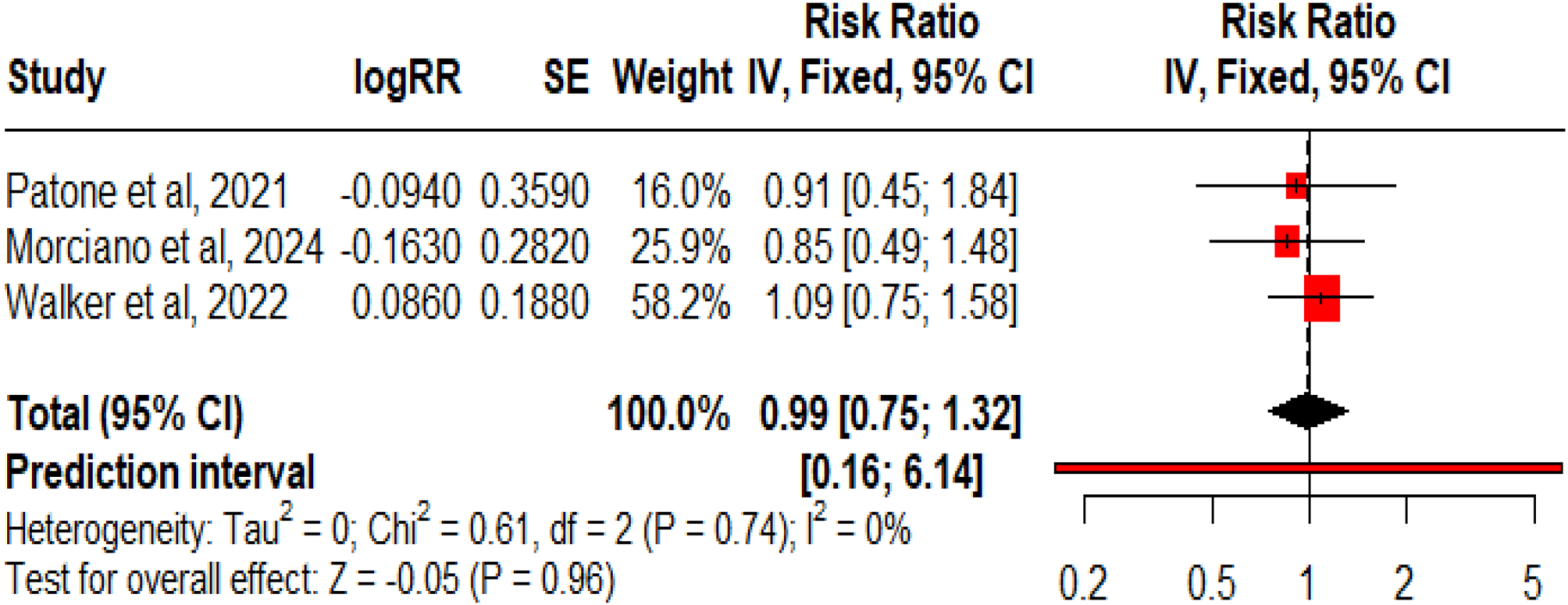
Forest Plot for RR for BNT162b2 mRNA vaccine.

For the BNT162b2 mRNA vaccine, we found 10 studies that reported on the vaccine’s association with GBS. Within them, 1609 cases were reported after administration of 916,053,583 doses of the vaccine. The reporting rate was 7.20 cases per million doses of the vaccine (Fig. 10). Figure 11 shows pooled RR from the 3 studies that reported it; there was no significant association noted between the *Pfizer-BioNTech (BNT162b2 mRNA) vaccine* and GBS incidence, RR: 0.99 (95% CI 0.75–1.32, p = 0.96). The funnel plot showed the presence of publication bias, but Egger’s regression test did not show the presence of publication bias.

### Moderna mRNA-1273 vaccine

**Figure 12:**
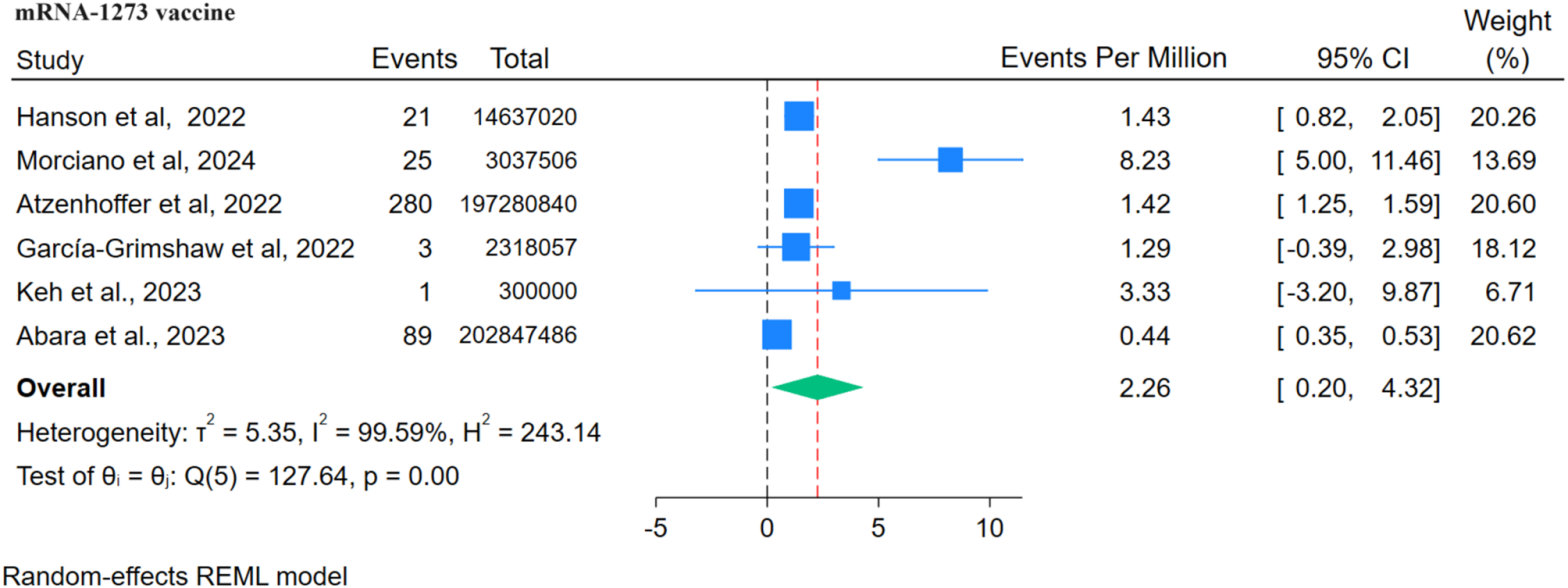
Forest Plots for GBS events per million doses of Moderna mRNA-1273 vaccine.

Six studies were found for the m-RNA-1273 vaccine, within these studies, 419 cases of GBS were reported after administering approximately 420,420,909 doses of the Moderna M RNA-1273 Vaccine. The reporting rate was 2.26 cases per million doses (Fig. 12). However, RR could not be analysed as only two studies were mentioned.

### Johnson and Johnson (Ad.26.COV2. S) vaccine

**Figure 13:**
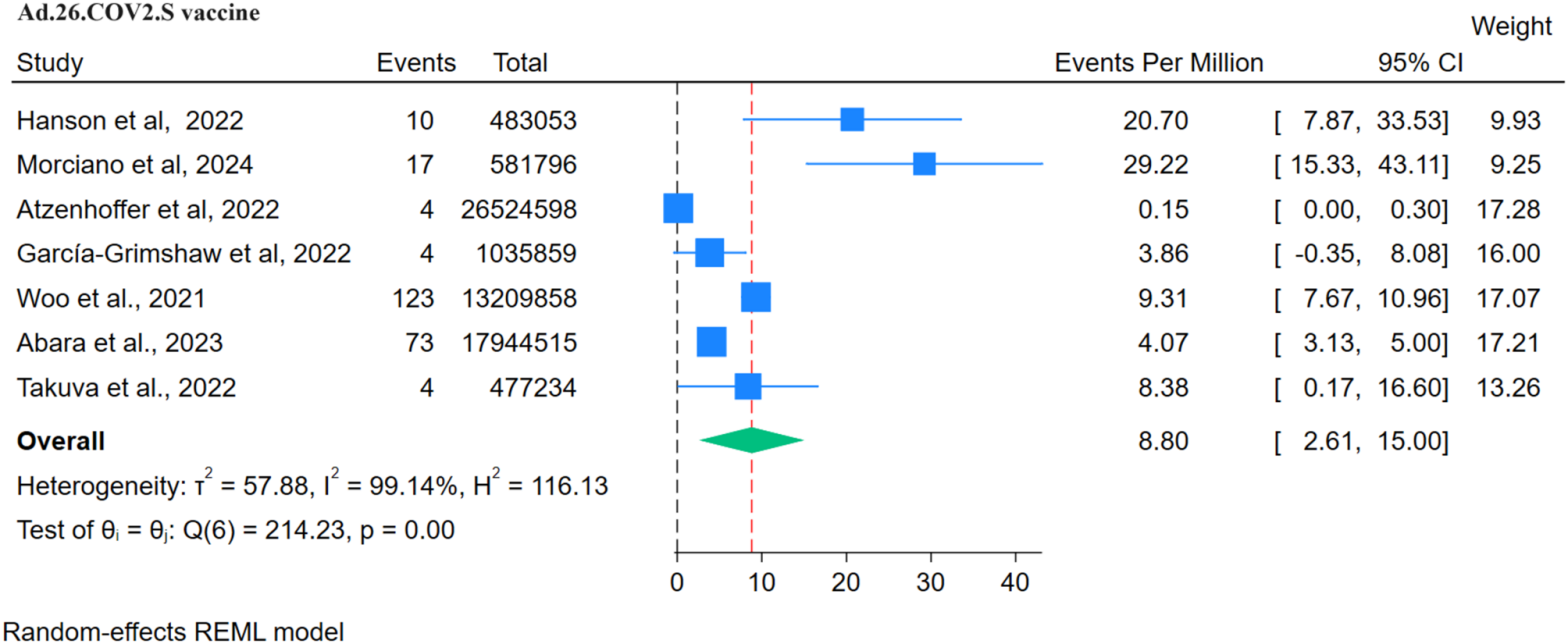
Forest Plots for GBS events per million doses for Ad.26.COV2. S vaccine.

**Figure 14:**
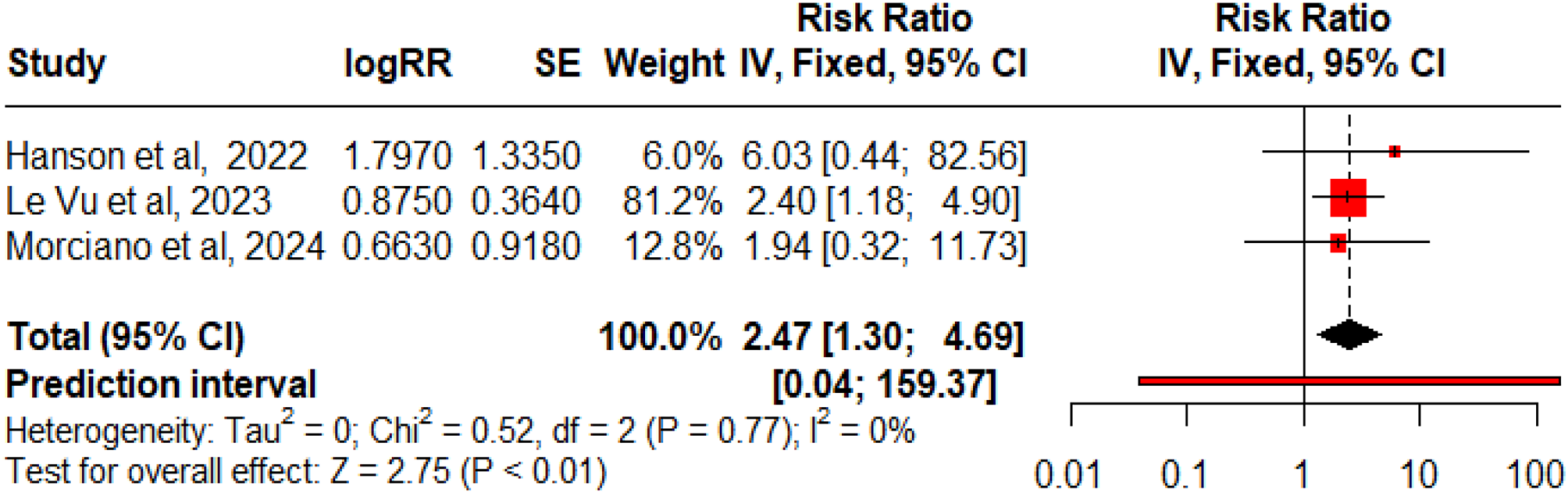
Forest Plot for RR for Ad.26.COV2. S vaccine.

Seven studies reported the impact of Ad.26.COV2. S vaccines in association with GBS. There were 235 GBS events recorded in these studies after the administration of 60,256,913 doses of the vaccine. The reporting rate was 8.80 per million doses (Fig. 13; Table 3). The RR from 3 studies was analysed using a fixed model since heterogenicity was not significant (Q: 0.52, p = 0.77, I^2^: 0%) (Fig. 14). A positive and significant association was seen between the risk of GBS and the administration of Ad.26.COV2. S vaccine, RR: 2.47 (95% CI 1.30–4.69, p < 0.01). The funnel plot showed the presence of publication bias, but Egger’s regression test did not show the presence of publication bias.

## Discussion

The COVID-19 vaccines demonstrated remarkable efficacy in preventing COVID-19 morbidity and mortality and have been the key milestone in the worldwide efforts to combat the COVID-19 pandemic^1^. However, the adverse neurological events following the COVID-19 vaccination have raised great concerns globally^31^. The present study results revealed a significant risk for GBS after the administration of the COVID-19 vaccine. The viral-vector vaccines were linked to higher GBS cases per million vaccine doses. Moreover, the GBS cases incidence after the first dose was higher than the second dose. The adverse events following COVID-19 vaccination are relatively rare but have been documented across the various vaccine platforms.

Bragazzi et al.^32^ conducted a study on the global burden of GBS and reported that the prevalence and disability of GBS have continued to escalate. There are about 150,095 total cases of GBS which resulted in 44,407 cases years lived with disability (YLDs) worldwide. Globally, there was a 6.4% increase in the prevalence of GBS per 100,000 population^32^. However, the COVID-19 pandemic further increased this incidence of GBS.

The manifestations of adverse neurological events following COVID-19 vaccination are diverse and may involve both central and peripheral nervous system dysfunction^29^. However, more severe neurological conditions, such as GBS, characterised by acute onset muscle weakness and sensory disturbances, have been reported following COVID-19 vaccines^33–36^.

GBS is an immune-mediated neurological condition characterized by swiftly developing ascending weakness, with sensory loss and nervous system involvement, leading to muscle weakness, paralysis, and sometimes life-threatening complications^37^. While the exact cause of GBS is not fully understood, it is triggered by infections or vaccinations and acute inflammatory demyelinating polyneuropathy. Several vaccines have been associated with GBS in the past. Therefore, there is a chance that the COVID-19 vaccines can also be associated with GBS^38^. The literature suggested the potential mechanisms are immune-mediated, the occurrence of GBS after the first dose of vaccines may cause an immune reaction sustained by later doses^39^. These findings need further clarification since the literature on the type of vaccine and recipient count is not available for comparison.

The recent studies examining the possible relationship between the COVID-19 vaccination and GBS have produced mixed findings. Some studies have reported a slightly elevated risk of GBS following vaccination, particularly with specific vaccine formulations^40^. However, other studies have found no increase in GBS risk associated with COVID-19 vaccination^41^. The global community concerns regarding the potential for COVID-19 vaccination to trigger GBS exist, but available evidence suggests that such events are exceedingly rare. Continued surveillance and research are necessary to understand any potential association better and ensure the safety of COVID-19 vaccination programs. Effective communication of risks and benefits is essential to maintain public trust and confidence in vaccination efforts amidst the ongoing pandemic.

The underlying molecular mechanisms of these adverse events of COVID-19 vaccines and GBS remain incompletely understood but are thought to involve immune-mediated processes, molecular mimicry, cross-reactive antibodies, or vaccine-induced inflammatory responses targeting the nervous system. The literature also highlights that anti-ganglioside antibodies and complement activation play a role in the pathogenesis of the GBS. The vaccine’s components can induce anti-ganglioside antibodies leading to inflammation. The immune cells produce antibodies against S-protein, which cross-reacts with gangliosides, and antibodies damage the neurons, thus leading to their demyelination^38,42^^.^

### Study strengths and limitations

Similar to other scientific studies, this study has strengths and limitations that must be considered while developing and implementing these results in any policy guidelines. This is the first study of its kind to explore the detailed analysis of four frequently used COVID-19 vaccines and their adverse effects on GBS.In this study, we analyzed the data regarding the type of vaccines, cases/events per million doses of vaccines, and their impact on GBS. Moreover, this study provided collective information on the adverse events of four frequently used vaccines on GBS. The study's limitations include the heterogeneity of the literature due to differences in study design, population, interventions, and outcomes measured. Moreover, detailed information about vaccination demographics is not fully available. Therefore, further studies are required to achieve better conclusions. Another limitation is that we did not use of safety signals protocol for causality assessment between adverse events following immunization to minimize the biases and confounders^43,44^.

## Conclusions

A positive and significant association was seen between the administration of the COVID-19 vaccine and the risk of GBS. The viral-vector vaccines are associated with 11.01 GBS cases per million vaccine doses. The incidence of GBS after the first dose was higher at 17.43 events per million compared to 1.47 cases per million in the second dose. The COVID-19 vaccination can be associated with GBS. Although the GBS cases after COVID-19 vaccination are relatively low, vaccination against COVID-19 is not to be delayed. The health officials must rule out the neurological manifestations associated with COVID-19 vaccination. Healthcare providers should remain vigilant in recognizing and managing these events, and ongoing surveillance and research are essential to elucidate the underlying mechanisms and optimize management strategies. Despite these challenges, the benefits of COVID-19 vaccination in mitigating the impact of the pandemic remain paramount, and efforts to address vaccine-related adverse events must be balanced with the imperative to achieve widespread immunization and control the spread of SARS-CoV-2.

### Supplementary Information

Supplementary Information.

Supplementary Information.

## Data Availability

The data may be provided on reasonable request to the corresponding author.
