# Supplementary material for: Exploring the adverse events of Oxford–AstraZeneca, Pfizer-BioNTech, Moderna, and Johnson and Johnson COVID-19 vaccination on Guillain–Barré Syndrome
Source: Sci Rep. 2024 Aug 13;14:18767. doi: 10.1038/s41598-024-66999-7 (PMC11322667; doi:10.1038/s41598-024-66999-7)
Supplement: Supplementary file 1 — Supplementary Information. [file 41598_2024_66999_MOESM1_ESM.docx]

**Supplementary material: Table 1: Eggers regression test**

|  | Intercept | p-value |
| --- | --- | --- |
| O/E analysis overall | 2.77 (1.50 4.05) | 0.000 |
| RR overall | 1.16031 (-.09 2.41) | 0.068 |
| O/E analysis m-RNA | 1.51 (-.39 3.40) | 0.1194 |
| O/E analysis viral-vector | 1.61 (-.15 3.24) | 0.06 |
| RR ChAdOx1nCoV-19 | 2.426 (0.82 - 4.03) | 0.21 |
| RR - BNT162b2 mRNA | -1.436 (-3.25 - 0.38) | 0.36 |
| RR- Ad.26.COV2.S | 0.445 (-0.95 - 1.85) | 0.65 |

**Supplementary material table 2: Leave-one-out sensitivity analysis**

|  | Omitted Study | Effect size with 95% CI | p-value |
| --- | --- | --- | --- |
| O/E analysis overall | Atzenhoffer et al, (France) 2022 (B+C) | 1.39 [0.79, 1.99] | -- |
|  | Atzenhoffer et al, (France) 2022 (A+D) | 1.28 [0.69, 1.86] | -- |
|  | Atzenhoffer et al, (Italy) 2022 (B+C) | 1.40 [0.82, 1.99] | -- |
|  | Atzenhoffer et al, (Italy) 2022 (A+D) | 1.33 [0.72, 1.93] | -- |
|  | Atzenhoffer et al, (USA) 2022 (B+C) | 1.38 [0.77, 1.98] | -- |
|  | Atzenhoffer et al, (USA) 2022 (A+D) | 1.20 [0.66, 1.75] | -- |
|  | Atzenhoffer et al, (Germany) 2022 (B+C) | 1.39 [0.79, 1.99] | -- |
|  | Atzenhoffer et al, (Germany) 2022 (A+D) | 1.23 [0.67, 1.79] | -- |
|  | Atzenhoffer et al, (Spain) 2022 (B+C) | 1.39 [0.80, 1.99] | -- |
|  | Atzenhoffer et al, (Spain) 2022 (A+D) | 1.29 [0.72, 1.86] | -- |
|  | Atzenhoffer et al, (Netherlands) 2022 (B+C) | 1.37 [0.77, 1.97] | -- |
|  | Atzenhoffer et al, (Netherlands) 2022 (A+D) | 1.29 [0.72, 1.86] | -- |
|  | Osowicki et al., 2022 (A+B) | 1.36 [0.76, 1.97] | -- |
|  | Woo et al., 2021 (D) | 1.10 [0.64, 1.57] | -- |
|  | Abara et al., 2023 (B) | 1.40 [0.81, 1.99] | -- |
|  | Abara et al., 2023 (C) | 1.40 [0.81, 1.99] | -- |
|  | Abara et al., 2023 (D) | 1.27 [0.68, 1.85] | -- |
|  | Li et al., 2022 (A) | 1.37 [0.77, 1.98] | -- |
|  | Li et al., 2022 (B) | 1.37 [0.76, 1.97] | -- |
| RR overall | Patone et al, 2021 (A) | 1.59 [0.93, 2.25] | 0.000 |
|  | Patone et al, 2021 (B) | 1.89 [1.17, 2.62] | 0.000 |
|  | Hanson et al, 2022 (C) | 1.93 [1.25, 2.61] | 0.000 |
|  | Le Vu et al, 2023 (A) | 1.67 [0.96, 2.37] | 0.000 |
|  | Le Vu et al, 2023 (D) | 1.72 [1.02, 2.42] | 0.000 |
|  | Morciano et al, 2024 (A) | 1.71 [1.05, 2.36] | 0.000 |
|  | Morciano et al, 2024 (B) | 1.91 [1.19, 2.63] | 0.000 |
|  | Morciano et al, 2024 (C) | 1.74 [1.08, 2.40] | 0.000 |
|  | Morciano et al, 2024 (D) | 1.76 [1.09, 2.43] | 0.000 |
|  | Walker et al, 2022 (A) | 1.56 [0.92, 2.20] | 0.000 |
|  | Walker et al, 2022 (B) | 1.88 [1.14, 2.62] | 0.000 |
| O/E m-RNA | Atzenhoffer et al, (France) 2022 | 0.36 [0.21, 0.52] | -- |
|  | Atzenhoffer et al, (Italy) 2022 | 0.40 [0.25, 0.54] | -- |
|  | Atzenhoffer et al, (USA) 2022 | 0.30 [0.20, 0.41] | -- |
|  | Atzenhoffer et al, (Germany) 2022 | 0.35 [0.20, 0.51] | -- |
|  | Atzenhoffer et al, (Spain) 2022 | 0.37 [0.22, 0.53] | -- |
|  | Atzenhoffer et al, (Netherlands) 2022 | 0.35 [0.21, 0.48] | -- |
|  | Osowicki et al., 2022 | 0.40 [0.25, 0.54] | -- |
|  | Abara et al., 2023 (B) | 0.39 [0.24, 0.54] | -- |
|  | Abara et al., 2023 (C) | 0.39 [0.23, 0.54] | -- |
|  | Li et al., 2022 | 0.36 [0.22, 0.50] | -- |
| O/E viral-vector | Atzenhoffer et al, (France) 2022 | 2.165 [1.401, 2.929] | -- |
|  | Atzenhoffer et al, (Italy) 202 | 2.312 [1.546, 3.078] | -- |
|  | Atzenhoffer et al, (USA) 2022 | 1.975 [1.324, 2.627] | -- |
|  | Atzenhoffer et al, (Germany) 202 | 2.046 [1.341, 2.751] | -- |
|  | Atzenhoffer et al, (Spain) 2022 | 2.134 [1.451, 2.817] | -- |
|  | Atzenhoffer et al, (Netherlands) | 2.136 [1.455, 2.817] | -- |
|  | Osowicki et al., 2022 | 2.241 [1.456, 3.025] | -- |
|  | Abara et al., 2023 | 2.169 [1.376, 2.963] | -- |
|  | Li et al., 2022 | 2.377 [1.814, 2.939] | -- |
| RR - ChAdOx1nCoV-19 | Patone et al, 2021 | 3.443 [0.795, 6.091] | 0.01 |
|  | Morciano et al, 2024 | 2.865 [2.385, 3.344] | < 0.00 |
|  | Walker et al, 2022 | 3.467 [0.888, 6.047] | 0.008 |
| RR - BNT162b2 mRNA | Patone et al, 2021 | 0.99 (0.68, 1.31) | 0.000 |
|  | Morciano et al, 2024 | 1.04 (0.69, 1.40) | 0.000 |
|  | Walker et al, 2022 | 0.87 (0.47, 1.27) | 0.000 |
| RR- Ad.26.COV2.S | Hanson et al, 2022 | 2.35 [0.55, 4.16] | 0.010 |
|  | Le Vu et al, 2023 | 1.96 [-3.70, 7.63] | 0.497 |
|  | Morciano et al, 2024 | 2.40 [0.50, 4.30] | 0.013 |

**Figures:**


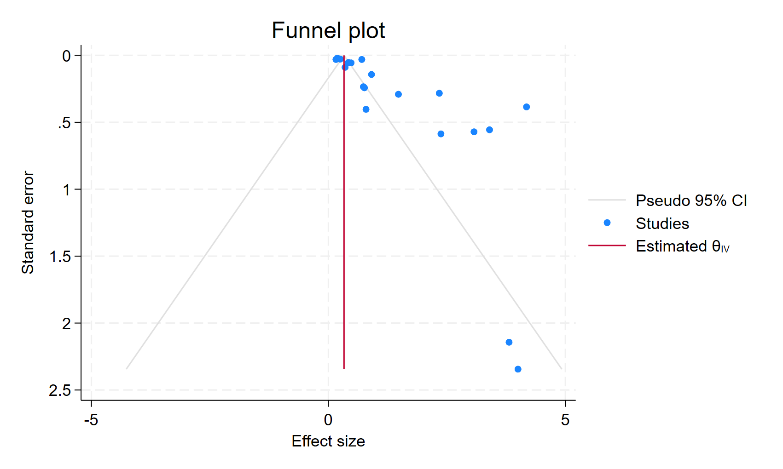


**Supplementary Figure 1: Funnel Plot for O/E overall**

**
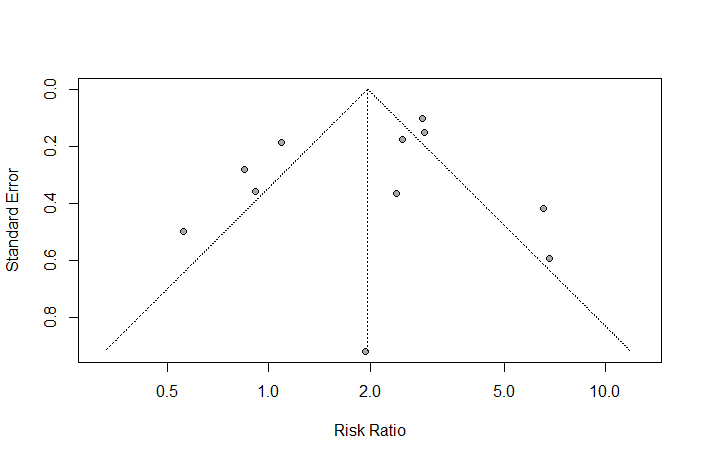
**

**Supplementary Figure 2: Funnel Plot for RR overall**

**
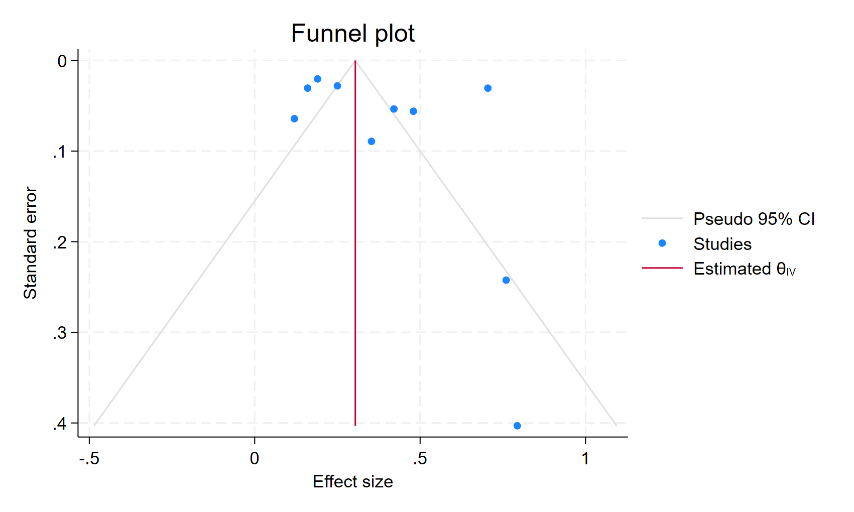
**

**Supplementary Figure 3: Funnel Plot for O/E m-RNA**

**
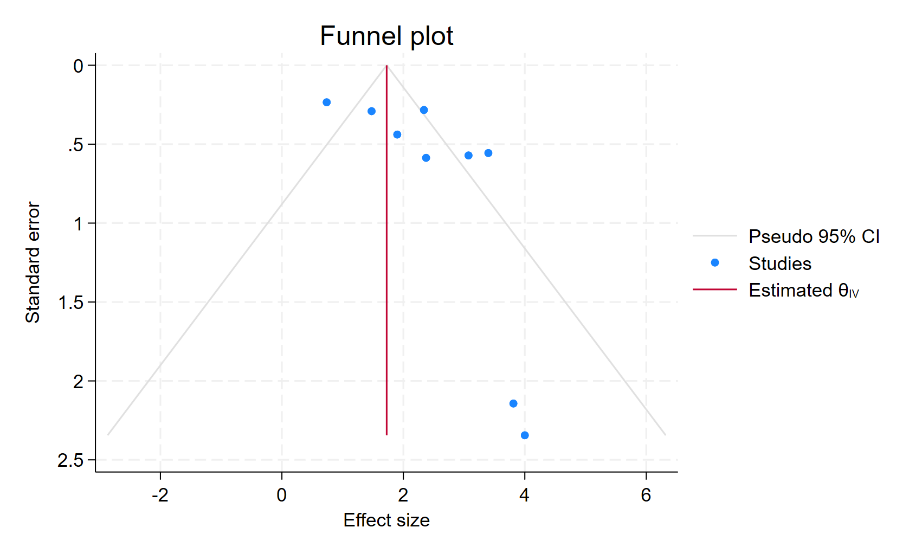
**

**Supplementary Figure 4: Funnel Plot for O/E viral-vector**

**
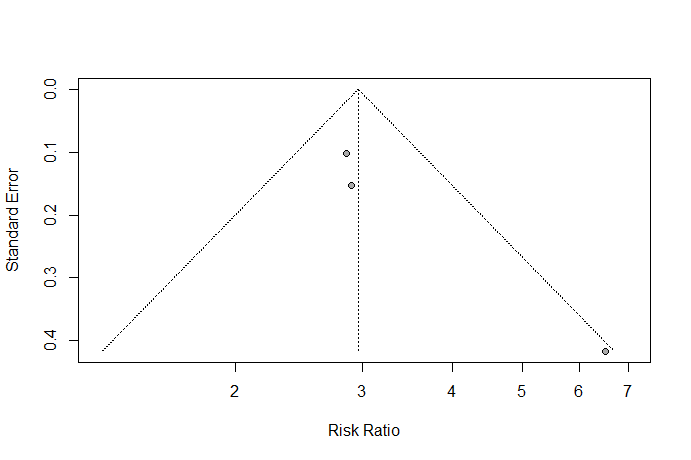
**

**Supplementary Figure 5: Funnel Plot for RR ChAdOx1nCoV-19**

**
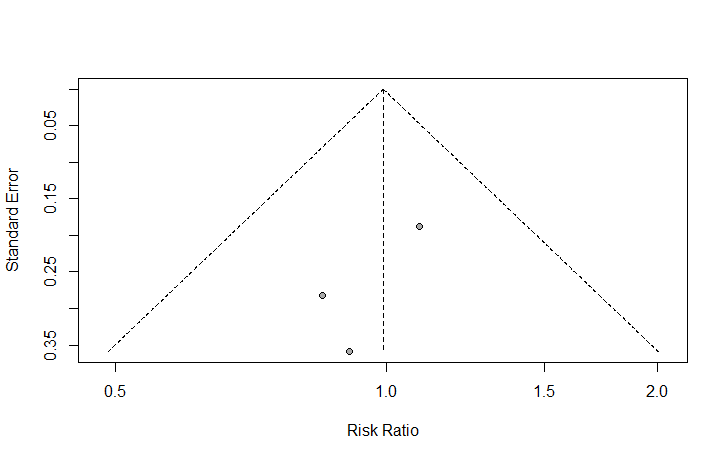
**

**Supplementary Figure 6: Funnel Plot for RR - BNT162b2 mRNA**


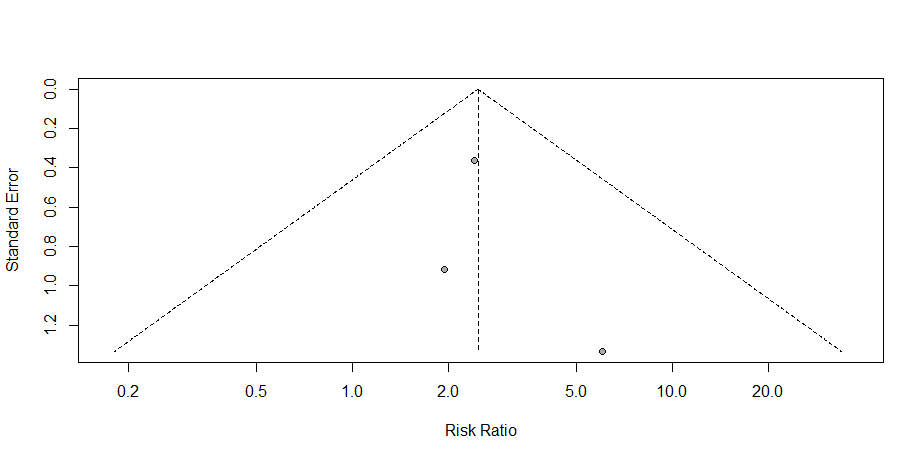


**Supplementary Figure 7: Funnel Plot for RR- Ad.26.COV2.S**
