# Supplementary material for: Exploring the adverse events of Oxford–AstraZeneca, Pfizer-BioNTech, Moderna, and Johnson and Johnson COVID-19 vaccination on Guillain–Barré Syndrome
Source: Sci Rep. 2024 Aug 13;14:18767. doi: 10.1038/s41598-024-66999-7 (PMC11322667; doi:10.1038/s41598-024-66999-7)
Supplement: Supplementary file 2 — Supplementary Information. [file 41598_2024_66999_MOESM2_ESM.docx]

Literature Search Strategy

| Search terms (AND, OR, NOT) and truncation (wildcard characters like *) | Oxford–AstraZeneca OR Pfizer-BioNTech OR Moderna OR Johnson and Johnson OR COVID-19 Vaccine* OR SARS-CoV-2 Vaccine* AND Guillain–Barré Syndrome OR GBS |
| --- | --- |
| Databases searched | Pub Med, Web of Science, Scopus, Google Scholar,  WHO, CDC, and Pharmaceutical Websites |
| Part of the journals searched | *Keywords in the abstract and title*  *MeSH terms* |
| Years of search | No years-based date restrictions were applied |
| Language | English language only |
| Types of studies to be included | *Original Articles*  Randomised-controlled trials (RCTs), Observational Studies (cohort, case-control and others) |
| Inclusion criteria (why did you include it?) | Types of Participants  1. Participants of any age who have received any of the following COVID-19 vaccines: Oxford–AstraZeneca, Pfizer-BioNTech, Moderna, or Johnson and Johnson.  2. Participants diagnosed with Guillain–Barré Syndrome (GBS) post-vaccination.  Types of Trials  Randomized controlled trials, and observational studies that investigate the association between COVID-19 vaccination and GBS.  Types of Interventions  Any study evaluating the adverse events of Guillain–Barré Syndrome, following COVID-19 vaccination.  Types of Outcomes  Primary Outcome: Incidence of Guillain–Barré Syndrome post vaccination. |
| Exclusion criteria (why did you rule it out? | Systematic reviews, brief communications, letters to the editor, case reports, and review articles |
